# A High-Throughput Method for Accurate Extraction of Intact Rice Panicle Traits

**DOI:** 10.34133/plantphenomics.0213

**Published:** 2024-08-01

**Authors:** Jian Sun, Zhengwei Ren, Jiale Cui, Chen Tang, Tao Luo, Wanneng Yang, Peng Song

**Affiliations:** National Key Laboratory of Crop Genetic Improvement, National Center of Plant Gene Research, Huazhong Agricultural University, Wuhan 430070, PR China.

## Abstract

Rice panicle traits serve as critical indicators of both yield potential and germplasm resource quality. However, traditional manual measurements of these traits, which typically involve threshing, are not only laborious and time-consuming but also prone to introducing measurement errors. This study introduces a high-throughput and nondestructive method, termed extraction of panicle traits (EOPT), along with the software Panicle Analyzer, which is designed to assess unshaped intact rice panicle traits, including the panicle grain number, grain length, grain width, and panicle length. To address the challenge of grain occlusion within an intact panicle, we define a panicle morphology index to quantify the occlusion levels among the rice grains within the panicle. By calibrating the grain number obtained directly from rice panicle images based on the panicle morphology index, we substantially improve the grain number detection accuracy. For measuring grain length and width, the EOPT selects rice grains using an intersection over union threshold of 0.8 and a confidence threshold of 0.7 during the grain detection process. The mean values of these grains were calculated to represent all the panicle grain lengths and widths. In addition, EOPT extracted the main path of the skeleton of the rice panicle using the Astar algorithm to determine panicle lengths. Validation on a dataset of 1,554 panicle images demonstrated the effectiveness of the proposed method, achieving 93.57% accuracy in panicle grain counting with a mean absolute percentage error of 6.62%. High accuracy rates were also recorded for grain length (96.83%) and panicle length (97.13%). Moreover, the utility of EOPT was confirmed across different years and scenes, both indoors and outdoors. A genome-wide association study was conducted, leveraging the phenotypic traits obtained via EOPT and genotypic data. This study identified single-nucleotide polymorphisms associated with grain length, width, number per panicle, and panicle length, further emphasizing the utility and potential of this method in advancing rice breeding.

## Introduction

Rice, one of the most widely grown crops in the world [1], plays a critical role in ensuring food security and socioeconomic stability [2]. Within the realm of rice breeding and cultivation, the pursuit of high-yielding, high-quality, and resilient varieties is a primary objective. Panicle traits, including the grain number, length, width, and panicle length, are vital for identifying rice germplasm resources and mapping functional genes [3]. These traits not only serve as indicators of yield potential but also play a crucial role in canopy architecture and photosynthesis [4,5]. Despite their importance, traditional manual measurement methods for assessing these traits are often laborious and time-consuming and require threshing, which can potentially damage grains and introduce measurement errors [6]. Thus, accurately and rapidly evaluating intact rice panicle traits is a significant challenge.

In recent years, the combination of computer vision and artificial intelligence technology has emerged as a transformative solution for crop phenomics [5]. In particular, deep learning has propelled the development of high-throughput phenotyping techniques. Various deep learning methods, including seed counting, size determination, and panicle segmentation, have shown impressive accuracy in crop trait detection. For instance, Wu et al. [7] utilized transfer learning with Faster R-CNN for wheat grain counting and calculated related traits in multiple scenarios, achieving an average accuracy of 91%. Quan et al. [8] leveraged a VGG19-based Faster R-CNN method to distinguish corn seedlings from soil and weeds, enhancing the accuracy to 97.7%. Hayat et al. [9] developed an unsupervised Bayesian learning algorithm for rice panicle segmentation, with an average *F*_1_ score of 82.1%. Misra et al. [10] introduced SpikeSegNet for wheat panicle detection, attaining a 95% accuracy rate in counting. These studies underscore the potential of deep learning in extracting rice panicle phenotypes.

Many researchers have focused on the phenotypic trait extraction of rice panicles after threshing. Zhao et al. [11] developed an image analysis method with a 5-point calibration model to count grains, achieving over 90% accuracy with less than a 10% error rate. Duan et al. [12] used visible light and soft x-ray imaging for grain number calculations. Liu et al. [13] created a mobile application for counting grains in rice and wheat, with an error rate below 2%. Tan et al. [14] introduced a counting algorithm that combined watershed and corner detection algorithms with neural network classification, achieving an average accuracy rate of 94.63%. However, the panicle threshing process can potentially damage rice grains [15], leading to inaccuracies in the final results. Therefore, it is crucial to perform trait extraction on intact rice panicles. To address this issue, Gong et al. [16] introduced a method that corrects the area and edge contour of rice panicles based on wavelet analysis. With this method, rice panicles were purposefully shaped before imaging, with each twig separated and affixed to a plate for accurate counting. An impressive average counting accuracy of 94% was achieved. Similarly, Lu et al. [17] proposed a high-precision analysis method using visible light scanning and deep learning, reaching an *R*^2^ value of 0.99 between the actual and detected grain number per panicle. Despite these advances, the artificial shaping required for these methods is time-consuming and costly, presenting barriers to large-scale phenotyping.

The panicle architectures of various rice varieties exhibit significant diversity, ranging from compact to loose structures. The main challenge in rice panicle phenotyping lies in devising a universally applicable model to ensure the efficient and accurate extraction of a wide array of panicle traits. Wu et al. [18] developed a method based on image analysis and deep learning that enables the rapid quantification of grain numbers per panicle in its natural morphology, with a statistical accuracy of 75%. In this study, we introduce a novel high-throughput method, termed extraction of panicle traits (EOPT), for assessing intact rice panicle traits, including grain number, grain length, grain width, and panicle length. The panicle morphology index (PMI) was developed to calibrate the impact of panicle architecture on counting accuracy. In addition, we integrate 2 novel trait extraction algorithms, one for panicle length analysis and the other for grain length and grain width extraction. By utilizing smartphones for image capture, EOPT circumvents the need for threshing and manual shaping.

## Materials and Methods

### Rice cultivation

The field experiments were conducted at Huazhong Agricultural University, located in Wuhan, Hubei Province, China (30.5°N, 114.3°E). This study utilized a total of 3,305 rice varieties obtained from the National Germplasm Resources Bank of the Chinese Academy of Agricultural Sciences in China. In the years 2022 and 2023, 2,840 and 45 varieties, respectively, were selected for continuous planting to assess year-to-year variability. Materials were allocated to individual plots, with the plots systematically organized into a grid pattern consisting of 5 rows and 4 columns. In 2023, a subset of 420 varieties was chosen from a pool of 533 varieties for a detailed analysis [19].

### Image acquisition

**Fig. 1.**
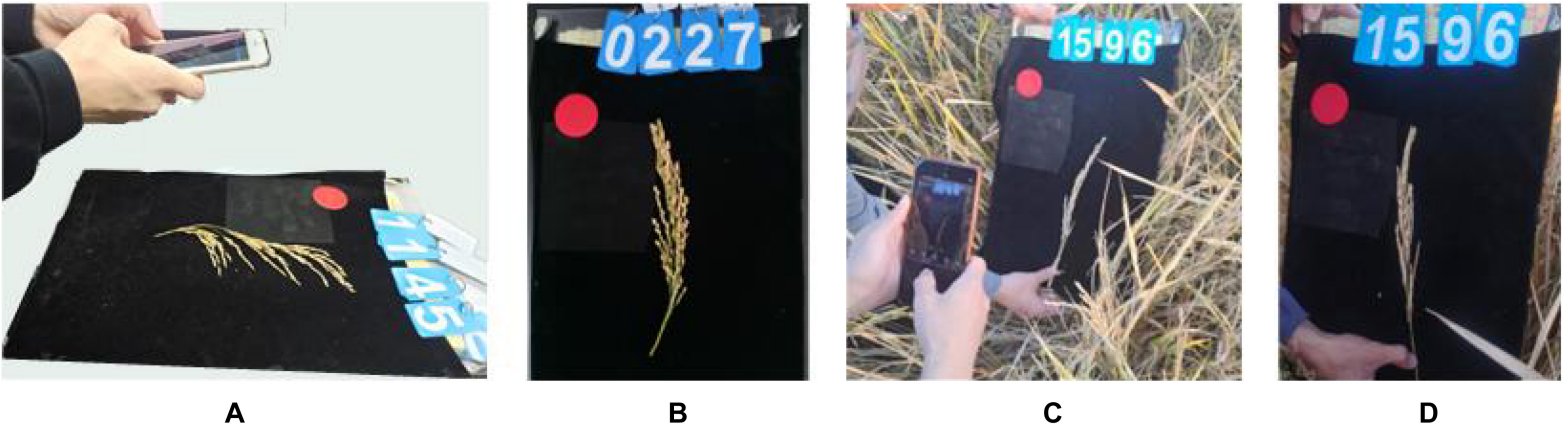
Rice panicle image acquisition. (A) Imaging of rice panicles in vitro. (B) Image of a rice panicle in vitro. (C) Imaging of rice panicles in the field. (D) Image of a rice panicle in the field.

Panicle images were captured using smartphones and with varying resolutions, primarily of 3,000 × 4,000 pixels and 2,736 × 3,648 pixels. The smartphones utilized in this experiment automatically adjusted the sensitivity and shutter speed. The acquisition protocol is depicted in Fig. 1A, where harvested panicles were positioned against a uniform background board. Images were taken approximately 35 cm away from the samples. A red calibration object was included in each image to facilitate subsequent distance calibrations during image analyses. The blue plates with numbers in Fig. 1 correspond to the planting numbers assigned to the respective rice varieties. These numbers were synchronized with the field planting records to maintain data consistency. Representative images of the collected rice panicles are displayed in Fig. 1B. Field images were also acquired, as shown in Fig. 1C. For these images, the background board was positioned laterally relative to the panicle to minimize background variability and shadow effects. A smartphone was used to photograph the panicles in situ, capturing the natural state of the rice panicles within their growing environment.

### Overview of the proposed method

**Fig. 2.**
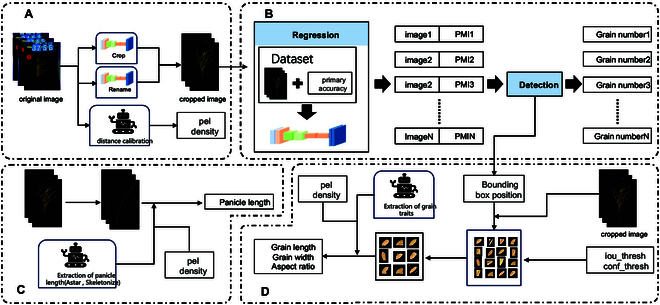
Flow diagram of the EOPT. (A) Image preprocessing. (B) Panicle grain counting module. (C) Panicle length extraction module. (D) Panicle grain length and width extraction module.

The proposed method for extracting rice panicle traits, termed EOPT, is depicted in Fig. 2. EOPT is an integrative image analysis approach that comprises 3 primary modules, a deep-learning-based module for grain counting (Fig. 2B), a module for extracting rice panicle length (Fig. 2C), and a module for extracting grain length and grain width (Fig. 2D).

The grain-counting module leverages deep learning techniques to enhance the accuracy of grain detection in intact panicles. A key feature of this module is the PMI, which is utilized to evaluate rice panicles based on their structural characteristics. The PMI is designed to mitigate the influence of the panicle structure on the counting accuracy that is typically encountered with grain counting methods by an object detection model. In the rice panicle length extraction module, the skeleton of the rice panicle is extracted to accurately trace the main stem route. This process involves the use of the Astar algorithm [20], which assists in delineating the central pathway of the rice panicle, ensuring precise measurement of its length. For the extraction of the grain length and grain width, appropriate confidence and intersection ratio thresholds are set to identify and segment grains with no occlusion or slight occlusion within the panicle image. The characteristics of these segmented grains are then used to represent the overall traits of the rice panicle.

All deep learning training for this experiment was run on the Ubuntu operating system (6-core i5 central processing units at 2.9 GHz per core, 12 GB of random-access memory, and an NVIDIA GeForce RTX 3080 graphics card). The CUDA 11.7 version was used for this experiment. The software used to train the model was PyCharm 2022.03.

### PMI definition

The accuracy of a grain counting method is significantly influenced by the panicle architecture, making it crucial to minimize the impact of morphology on the counting results. Rice panicle architectures exhibit considerable diversity, with structures ranging from compact to loose. The primary challenge in phenotyping rice panicles lies in developing a universal model that ensures efficient and accurate extraction of the panicle traits. In loose panicle structures, the grains are prominently visible with minimal occlusion in the images. Conversely, compact panicle structures present significant mutual occlusion, leading to many grains being obscured in images.

Traditional object detection algorithms struggle to account for the obscured portions of grains in compact panicles, causing inaccuracies in grain counting. To address this, we propose the use of a PMI to adjust the counting results of the traditional object detection methods to enhance their accuracy. The PMI is defined on the basis of the correlation between the rice panicle structure and its traits. It describes the degree of mutual occlusion among the grains within the panicle. The PMI ranges from a minimum value of 0 to a maximum value of 1. A PMI closer to 1 indicates minimal mutual occlusion among the panicle grains, while a PMI closer to 0 indicates severe occlusion among the grains within the rice panicle.

An image regression network was designed to address the numerical regression challenge posed by rice panicle structures. Initially, we calculated the PMI on a set of 4,000 rice panicle images. In this study, the predicted value of the number of grains for each image of a rice panicle was first obtained by the grain detection model. The true value of each rice panicle sample was obtained by manual measurement. The PMI of each panicle was obtained by substituting the predicted and true values into Eq. 1. Subsequently, we constructed a dataset of the panicle images and their corresponding PMIs. The dataset is trained using the proposed model. A neural network that can predict PMI based on images is obtained.

**Fig. 3.**
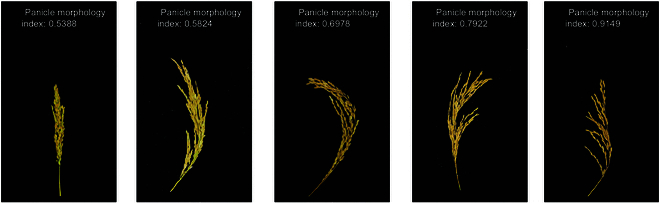
PMI of rice panicles with different structures.

The model can infer the corresponding PMI based on the rice panicle image. The more severe the grain occlusion is, the lower the calculated PMI. Figure 3 displays the PMI for images of panicles with various architectures. The true value of the PMI is calculated as follows:

$$\begin{array}{c} \text{PMI}=1-|\;\left(N_{\text{actual}}-N_{\text{forecast}}\right)/N_{\text{actual}}\;| \end{array}$$ (1)

where *N*_actual_ represents the actual grain number of a panicle and *N*_forecast_ represents the predicted grain number of a panicle.

**Fig. 4.**
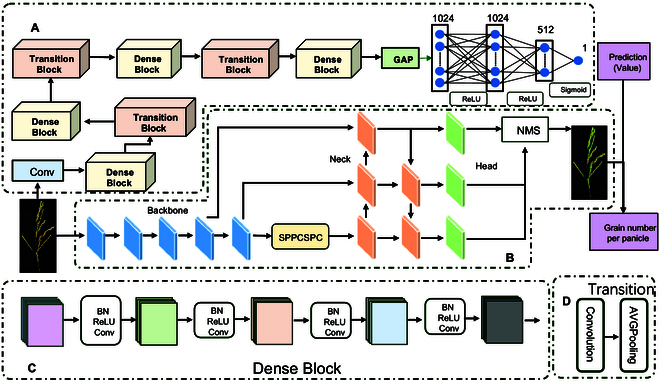
Flow chart of the grain counting model. (A) The structure of the PMI calculation module. (B) The structure of the counting module. NMS, nonmaximum suppression. (C) The structure of the dense block. (D) The structure of the transition block.

The network structure of the PMI calculation module is shown in Fig. 4A. The fully connected layer of the DenseNet121 model [21] is removed for feature extraction, and a global average pooling layer compresses the feature map into a 1,024-length one-dimensional vector. The network structure primarily comprises DenseBlock [22] and transition layers (Fig. 4C and D). In DenseBlock, the feature map size of each layer is consistent. Here, DenseNet proposes a dense connectivity mechanism that connects all the layers to each other; specifically, each layer accepts all the layers before it as its additional input. Each layer is connected (concatenated) with all previous layers in the channel dimension and used as input for the next layer. This enables feature reuse and improves efficiency. The nonlinear combination function H, used in DenseBlock, adopts the BN (batch normalization) + ReLU (rectified linear unit) + 3 × 3 Conv structure. For the transition, it primarily connects 2 adjacent DenseBlocks and reduces the feature map size. The transition layer includes a 1 × 1 convolution and 2 × 2 AvgPooling layer, with a BN + ReLU + 1 × 1 Conv + 2 × 2 AvgPooling structure.

Leveraging the DenseNet121 model, the network uses transfer learning to extend the pretrained model’s performance to additional datasets. The DenseNet-1k model, pretrained on ImageNet [23] without a fully connected layer, is used for this purpose. In Fig. 4A, we use DenseNet121 as the backbone and incorporate a global average pooling layer [24] to reduce the feature dimensions. After processing by the global average pooling layer, a feature vector with a length of 1,024 is obtained. This feature vector is subsequently processed to obtain the final output by means of a regression header, which consists of 3 fully connected layers. Before inputting images into the model, we preprocessed them as follows. The main portion of the rice panicle was identified using object detection, and on the basis of these results, images were cropped to retain the main part of the rice panicle. Cropped images were then padded using black pixels to ensure a uniform aspect ratio across all images. After observing the size of the cropped images, the most distributed size was 1,800 × 1,100 (with an aspect ratio of 1.666); thus, we standardized the aspect ratio to 1.666. All the images were resized to 900 × 550. For network training and testing, we utilized the mmpretrain open-source deep learning framework to address the numerical regression challenges of the rice panicle structures. The learning rate is modulated using MultiStepLR with a factor of 0.1, and the network uses the stochastic gradient descent optimizer with a momentum of 0.9.

### Grain counting of intact panicles using PMI

The network structure of the counting module is presented in Fig. 4B. It is first activated by 4 cross-stage partial network modules for convolution, batch normalization, and the LeakeyReLU function. Then, features are alternately extracted by the efficient layer attention network (ELAN) module and the max pooling module. Finally, the outputs of 3 ELAN modules are used as inputs to the head. The ELAN module is an efficient network structure that enables the network to learn more features by controlling the shortest and longest gradient paths, and it has strong robustness. The max pooling module first performs downsampling and then fuses the sensory field expansion obtained through the MaxPooling operation with the feature information from normal convolutional processing to improve the generalizability of the network. The detection head uses the ELAN module and the spatial pyramid pooling and cross-stage partial connections (SPPCSPC) module to aggregate the image features, and then the Rep module adjusts the output detection results. The SPPCSPC module first divides the features into 2 parts. One part is routinely processed, and the other part uses multiple maximum pooling to obtain different receptive fields; this is done so that the algorithm adapts to images with different resolutions. Finally, the 2 parts are combined to reduce the amount of computation and improve the model detection accuracy. In the output of the network, the position of each detection box is encoded as an offset relative to the feature map, with confidence scores indicating target presence. The model is trained and validated using the Python programming language. The computational framework is based on the PyTorch deep learning library, utilizing CUDA version 11.7. The key hyperparameters include a batch size of 4, a learning rate of 0.01, and a maximum of 500 epochs. The stochastic gradient descent optimizer is used during training with a momentum of 0.937. After obtaining the PMI and initial number of grains, we used the following formula to calculate the final number of grains per panicle:

$$N_{\text{grain}}=N_{\text{predict}}/\text{PMI}$$ (2)

where *N*_grain_ represents the final number of grains in the panicle, *N*_actual_ represents the actual value, and *N*_forecast_ represents the predicted value.

The mean average precision at the 50% threshold (mAP_50_), mean absolute error (MAE), root mean square error (RMSE), mean absolute percentage error (MAPE), and coefficient of determination (*R*^2^) are used to evaluate the results of the model. The evaluation index formula is as follows:

$$\text{MAPE}=\left(1/n\right)\;\times \;\Sigma \left(\;|\;\left(N_{\text{actual}}-N_{\text{forecast}}\right)/N_{\text{actual}}\;|\right)\;\times \;100\%$$ (3)

$$\text{RMSE}=\surd \left(\left(\Sigma {\left(N_{\text{actual}}-N_{\text{forecast}}\right)}^{2}\;\right)⁄n\right)$$ (4)

$$\text{MAE}=\left(1/n\right)\;\times \;\Sigma \left(|\;\;N_{\text{actual}}-N_{\text{forecast}}\;|\right)$$ (5)

$$R^{2}=1-\Sigma \left(\;\;{\left(N_{\text{actual}}-N_{\text{forecast}}\right)}^{2}\right)/\Sigma \left(\;\;{\left(N_{\text{actual}}-<N_{\text{actual}}>\right)}^{2}\right)$$ (6)

$$\text{Accuracy}=\left(1-\text{MAE}/N_{\text{actual}}\right)\;\times \;100\%$$ (7)

where *n* represents the number of samples, *N*_actual_ represents the actual value, *N*_forecast_ represents the predicted value, and <*N*_actual_> indicates the average of the predicted values.

### Grain length and grain width extraction

In this study, we develop a method for determining rice grain length and width, which leverages the panicle grain counting model to provide positional information about the grains within the panicle. The model generates anchor boxes lacking in orientation, and the inclination angle and degree of occlusion of the grains can lead to significant errors in measuring grain traits. To overcome this, we devised a novel strategy for the extraction of grain lengths and widths.

Our strategy’s process is as follows. First, we use the grain counting model to predict the images and collect individual grain images. We then select these grains based on 2 parameters, the intersection over union (IoU) and confidence threshold (Conf), to identify grains with no occlusion or slight occlusion. Next, we calculate the smallest outer rectangle aligned with the grain orientation of the selected grains to accurately extract the grain length and width. Here, the IoU was used to measure the overlap between the predicted and actual bounding boxes, with the IoU ranging from 0 (no overlap) to 1 (perfect overlap). The Conf was set to determine whether a predicted box contained the target, with boxes exceeding this threshold deemed positive samples. A predicted box that is too small can lead to incomplete grains after cropping. Therefore, we compared the effects of cropping using the original size and cropping after padding 2 pixels (Table 1).

**Table 1.** Results of various cropping methods

Thresholds	Segmentation method	*R*^2^ of grain length	*R*^2^ of grain width	*R*^2^ of aspect ratio
Conf 0.45 IoU 0.8	Original size	0.77	0.33	0.77
Conf 0.5 IoU 0.75	Original size	0.77	0.33	0.77
Conf 0.8 IoU 0.8	Original size	0.86	0.27	0.77
	Padding 2 pixels	0.84	0.32	0.79
Conf 0.7 IoU 0.8	Original size	0.86	0.28	0.78
	Padding 2 pixels	0.84	0.33	0.79

In this experiment, we conducted separate croppings of grain images using various combinations of IoU and Conf after detecting grains. Subsequently, we measured the grain lengths and widths in these cropped images to determine the optimal IoU and Conf combination. The results of the various cropping methods are shown in Table 1. The best outcomes were observed with an IoU threshold of 0.8 and a Conf of 0.7 under the original image size, resulting in *R*^2^ values of 0.86 and 0.78 for the measured versus true values of the grain length and aspect ratio, respectively.

**Fig. 5.**
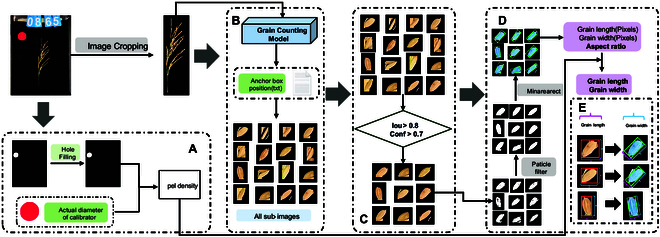
Flow chart of the grain length and grain width extraction algorithm. (A) Distance calibration. (B) Process of grain image cropping. (C) Grain screening. (D) Grain trait extraction algorithm. (E) Comparison of the original frame and the proposed method frame.

Grain traits were extracted after the selected grain areas were segmented, and the extraction process is illustrated in Fig. 5. The procedure begins with threshold segmentation and particle filtering to extract the main body of the grain. Then, the minimum bounding rectangle of the grain’s main body is identified, with its dimensions recorded as the grain’s length and width. Despite our initial use of a fixed shooting distance during image capture, EOPT does not require a fixed distance for practical application. This is because EOPT calculates the real-world size represented by each pixel using a calibration object. In this calibration process, depicted in Fig. 5A, we compute the “pel density”, which indicates the real-world size corresponding to each pixel in the image. By analyzing the red calibrator’s radius in pixels and setting the real-world radius of the red calibrator to 25 mm, we calculate the pel density as 25 divided by the calibrator’s pixel radius. This calculation enables us to obtain the true values of the grain length, width, and aspect ratio.

### Panicle length extraction

**Fig. 6.**
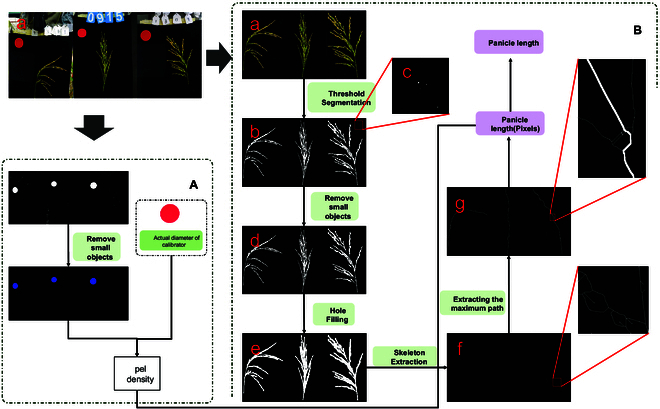
Flowchart of the panicle length extraction algorithm. (A) Distance calibration. (B) Panicle length extraction algorithm. (a) The original image. (b) The binary image. (c) Noise in the enlarged image. (d) The image after removing small objects. (e) Image after hole filling. (f) Image after skeletonization. (g) Image of the main pathway of the rice panicle.

Measuring the panicle length of rod-shaped rice panicles is relatively straightforward; however, determining the panicle length of loose rice panicles presents a complex challenge due to their structure. To address this issue, we developed a method that allows for the rapid and accurate extraction of panicle lengths from images. The panicle length extraction method process is shown in Fig. 6. Initially, we perform threshold segmentation on the S-component of the panicle image. In the hue, saturation, and value color space, the S-component represents saturation, and the S-component image is also known as a grayscale image representing the saturation of each pixel. This step segments panicles from the background. Subsequently, small areas are removed to eliminate noise, impurities, and any calibration objects. Next, any holes in the image are filled to smooth the rice panicle silhouette, which is crucial for skeleton extraction. We use the skeletonize function from the skimage library [20], which offers a more efficient alternative to traditional thinning methods, thus significantly accelerating the computation process. Once the skeleton is extracted, all the end points are identified. An end point is defined as a pixel that has only one adjacent foreground pixel within its 8-pixel neighborhood. Then, the Astar algorithm [21] is used for path planning to delineate the main path of the panicle.

The extraction strategy involves designating the bottom end point as the starting point and the top 20 end points as potential end points. The shortest path between the starting point and each potential end point is calculated on the basis of the number of pixels it encompasses. The path with the highest pixel count is considered the main path of the rice panicle. The calibration process for determining the real-world size corresponding to each pixel is illustrated in Fig. 6A. Threshold segmentation is performed on the original image to obtain the dimensions of the calibration object. With the actual known size of the calibration object, we calculate the actual size represented by each pixel. Finally, this information is used to compute the real-world panicle length.

### EOPT-based software development: Panicle Analyzer

**Fig. 7.**
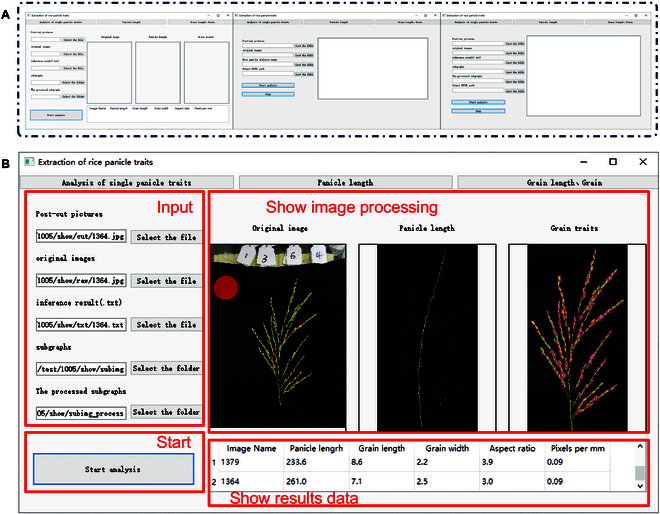
Interface of the Panicle Analyzer. (A) Interfaces with different functions. (B) Results of the main functional interface are displayed.

In this study, we develop a PC-based software application named “Panicle Analyzer”. This software is designed to extract phenotypic traits from images of rice panicles captured by smartphones. This software facilitates the analysis of panicle characteristics by providing an intuitive user interface and automated trait extraction capability. The main user interface of the software, presented in Fig. 7A, is organized into 3 main sections, which are, arranged from left to right, a trait extraction module for individual panicles, a batch processing module for panicle length, and a batch processing module for grain length and width. These modules enable users to process images, review results, and export data efficiently. The Panicle Analyzer processes the input images and displays both the original and processed images, along with the extracted trait data. The processed images include the skeletonized representation of the panicle and the segmented grain images (Fig. 7B). The software displays various data, including the ID of the panicle image, the measured panicle length, grain length and width, aspect ratio, and pixel density, which are calibrated for distance. The extracted traits are compiled and saved in an Excel file, allowing for easy data management and further analysis, providing a convenient format for researchers to access and utilize the data. The link for the project is https://github.com/SUNJHZAU/EOPT. The link for the software is https://pan.baidu.com/s/1BJ184slKAXJMwLmZznjRlA?pwd=6knk.

### A genome-wide association study validates the practicality of EOPT

In 2023, we cultivated 420 rice varieties. For each variety, we collected images from 4 individual samples. These images were used to extract the phenotypic traits of the rice panicles via the EOPT method. The trait values for each rice material were averaged to obtain a representative value for subsequent analysis.

To investigate the genetic basis of the extracted phenotypic traits, we performed a genome-wide association study (GWAS) using FaST_LMM software. A mixed linear model was applied, which included kinship as a random effect to account for potential relatedness among the rice varieties. The association between genome-wide single-nucleotide polymorphism (SNP) markers and phenotypic variation was assessed to identify the genetic loci associated with the traits of interest.

SNP sites with a minor allele frequency of less than 5% were excluded from the analysis. After filtering, a total of 4,358,600 high-quality SNP sites were included in the study. The significance threshold for SNP relatedness set in this study was 1/4,358,600. The strength of the association between SNPs and phenotypic traits was visualized using a Manhattan plot. In addition, a quantile–quantile plot was constructed to evaluate the model’s effectiveness in controlling for population structure and other confounding factors. To identify potential candidate genes, we searched within a 100-kb window up- and downstream of significant SNPs utilizing the rice genome annotation file for reference.

## Results

The study included a total of 3,305 rice varieties. In 2022, we collected 5,554 images from 2,840 varieties. These images were utilized for different purposes: 4,000 images were used to construct a grain counting model for intact rice panicles; 1,149 images were used to validate the accuracy of the grain counting model; 359 images were used to assess the accuracy of EOPT for grain length and width; and 46 images were used to evaluate the accuracy of EOPT for measuring panicle length. In 2023, we focused on 45 varieties, capturing 45 images indoors and an additional 45 images in the field. The indoor images were used to validate the performance of EOPT across different years, ensuring the method’s consistency over time. Conversely, the field images were intended to test the adaptability of EOPT to field conditions, demonstrating its practical applicability. For the other 420 varieties cultivated in 2023, we amassed a total of 1,680 images. These images were analyzed to perform a GWAS, aiming to link the phenotypic traits extracted by EOPT with genetic markers. Details of the samples used in this study are summarized in Table 2.

**Table 2.** Image and variety used in the experiments

Acquisition date	Varieties number	Imaging environment	Images number	Application
October 2022	2,840	Indoor	4,000	Training the grain counting model
			1,149	Testing the effect of the grain counting model
			359	Verification of the extraction effect of grain length and width
			46	Validating the effect of panicle length extraction
September 2023	420	Indoor	1,680	GWAS analysis
October 2023	45	Indoor	45	Effectiveness of validation methods in different years
		Outdoor	45	Validation of the adaptability of the method in the field

### Evaluation of rice panicle grain counts

**Fig. 8.**
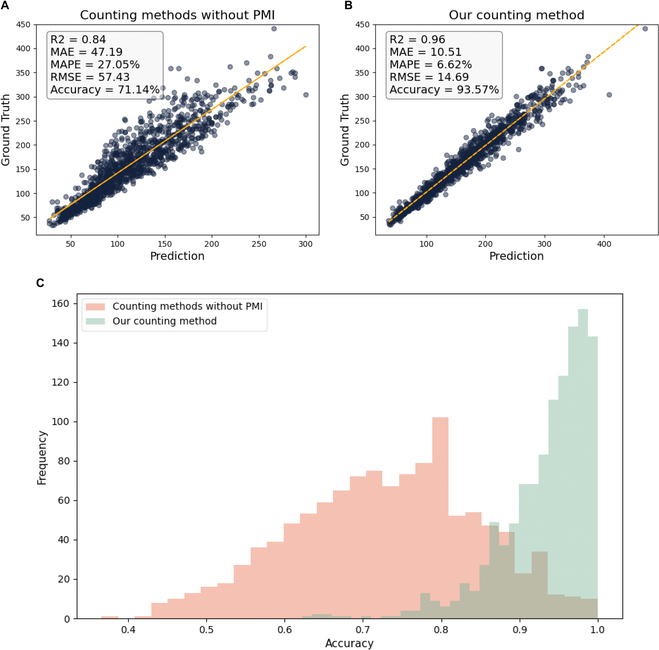
Comparison of the effects of different methods on the grain count of rice panicles. (A) The prediction results of the counting method without the PMI. (B) The prediction results of our counting method. (C) The distribution of the accuracy between different methods for counting rice grains.

This study evaluated the rice panicle grain model using a dataset of 1,149 rice panicle images. The correlation between the number of grains detected by traditional methods and the actual grain count was represented by a coefficient of determination (*R*^2^) of 0.84 (Fig. 8A). This significant correlation suggests a strong relationship between the detected and real values. However, the counting accuracy of the method without PMI was 71.14%, with an MAE of 47 grains and an MAPE of 27.05%, indicating a relatively high level of error. These results demonstrate the limitations of traditional methods for accurately counting grains in intact rice panicles. We further evaluated the prediction results of the PMI, where the *R*^2^ value between the predicted and true grain counts was 0.74, and the prediction accuracy reached 93.61%. The MAE, MAPE, and RMSE were 4.66%, 6.59%, and 6.05%, respectively. This indicates that the proposed method can accurately predict PMI. The results of the grain counting method proposed in this study are presented in Fig. 8B. Compared to that of the traditional method, the counting accuracy of the EOPT improved from 71.14% to 93.57%. The coefficient of determination (*R*^2^) between the predicted and true values increased from 0.84 to 0.96. The MAE decreased from 47.19 to 10.51, and the MAPE decreased from 27.05% to 6.62%. As shown in Fig. 8C, the accuracy of the traditional method for directly counting grains in natural panicle morphology was mostly below 90%; however, the accuracy of the proposed grain counting model was predominantly above 90%. These findings suggest that the EOPT can be used to accurately measure the number of grains in a rice panicle in its natural morphology and that the use of PMI effectively mitigates the impact of morphological differences in rice panicles on the counting results.

### Evaluation of grain width and grain length

We assessed the efficacy of EOPT for determining grain length and width using a dataset of 359 rice panicle images. The validation results are summarized in Table 3. The EOPT method demonstrated high accuracy in measuring grain length (96.83%), grain width (91.56%), and aspect ratio (89.92%). The MAPE for grain length, grain width, and aspect ratio were all less than 10%. The findings suggest that EOPT is capable of accurately extracting information about traits such as grain length and width from rice panicle images. Moreover, the traits of a subset of complete grains were found to be representative of the entire grain set, offering a practical approach for phenotypic analysis. This study not only confirms the reliability of EOPT for rice phenotyping but also introduces a novel perspective for trait extractions in other crop species.

**Table 3.** Results of grain length and width extraction

Traits	MAE	MAPE	RMSE	Accuracy
Grain length	0.240	3.08%	0.339	96.83%
Grain width	0.244	8.78%	0.307	91.56%
Aspect ratio	0.275	9.99%	0.359	89.92%

### Evaluation of panicle length extractions

To assess the accuracy and robustness of the panicle length extraction algorithm, we analyzed 46 rice panicle images. All 46 panicle images were collected indoors. The images were captured under various camera parameters and illumination conditions. This variability in image acquisition was used to test the adaptability of the algorithm across a range of scenarios. The *R*^2^ between the algorithm-predicted panicle lengths and their actual measurements was 0.953, indicating a high degree of correlation. The algorithm exhibited an impressive accuracy rate of 97.13% for panicle length measurements. In addition, an MAE of 0.71 cm was recorded, and the MAPE was 2.79%. These results substantiate the ability of EOPT to measure the length of rice panicles with high accuracy. The low MAE and MAPE values further confirm the effectiveness of the algorithm, even when it is subjected to various imaging conditions, thus validating its practical application for accurate phenotypic measurements of rice panicles.

### Validation of EOPT adaptability

**Fig. 9.**
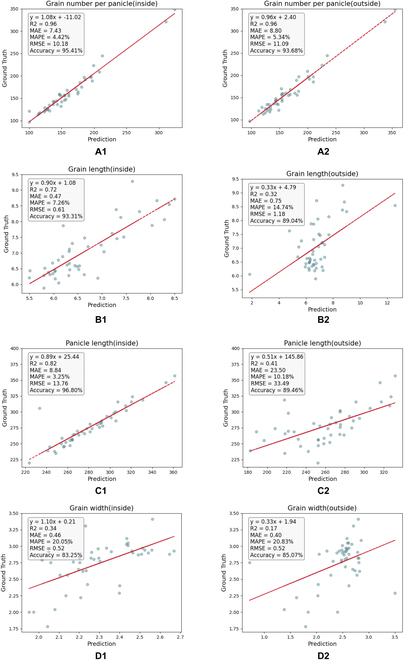
Results of validation of EOPT. (A1) Panicle grain number detection indoors. (A2) Panicle grain number detection in the field. (B1) Grain length detection indoors. (B2) Grain length detection in the field. (C1) Indoor panicle length detection. (C2) Panicle length detection in the field. (D1) Grain width detection indoors. (D2) Grain width detection in the field. The units for all traits except grain number per panicle are millimeters.

A diverse collection of rice varieties was selected to evaluate the universality and applicability of EOPT. Images were collected both indoors and outdoors to assess the performance of EOPT under various conditions. As illustrated in Fig. 9, the results of the traits extracted using EOPT from indoor rice panicle images achieved an accuracy of 95.41% for counting the number of grains per panicle, with an MAPE of 4.42%. In addition, the accuracies for the extraction of the grain length, width, and aspect ratio were 93.31%, 83.25%, and 85.57%, respectively, with corresponding MAPEs of 7.26%, 20.05%, and 13.21%, respectively. Panicle length extraction demonstrated an accuracy of 96.80%, an MAE of 8.84 mm, and an MAPE of 3.25%. These results indicate that EOPT can be used to consistently and accurately measure the traits of intact rice panicles across different years.

Measuring traits on intact rice panicles is necessary in certain scenarios, such as for studying circadian rhythms or examining growth and development during the grain-filling period. A field image analysis revealed an accuracy of 93.68% for counting the number of grains per panicle, with an MAPE of 5.34%. The accuracies for extracting grain length, width, and panicle length were 89.04%, 85.07%, and 89.46%, respectively. Although the accuracies for grain length and panicle length measurements in the field environment were slightly lower than those obtained from detached panicle images, the overall high accuracy and low error margins affirm that EOPT reliably extracts panicle phenotypic traits in the field.

### Results of genome-wide association analysis

**Fig. 10.**
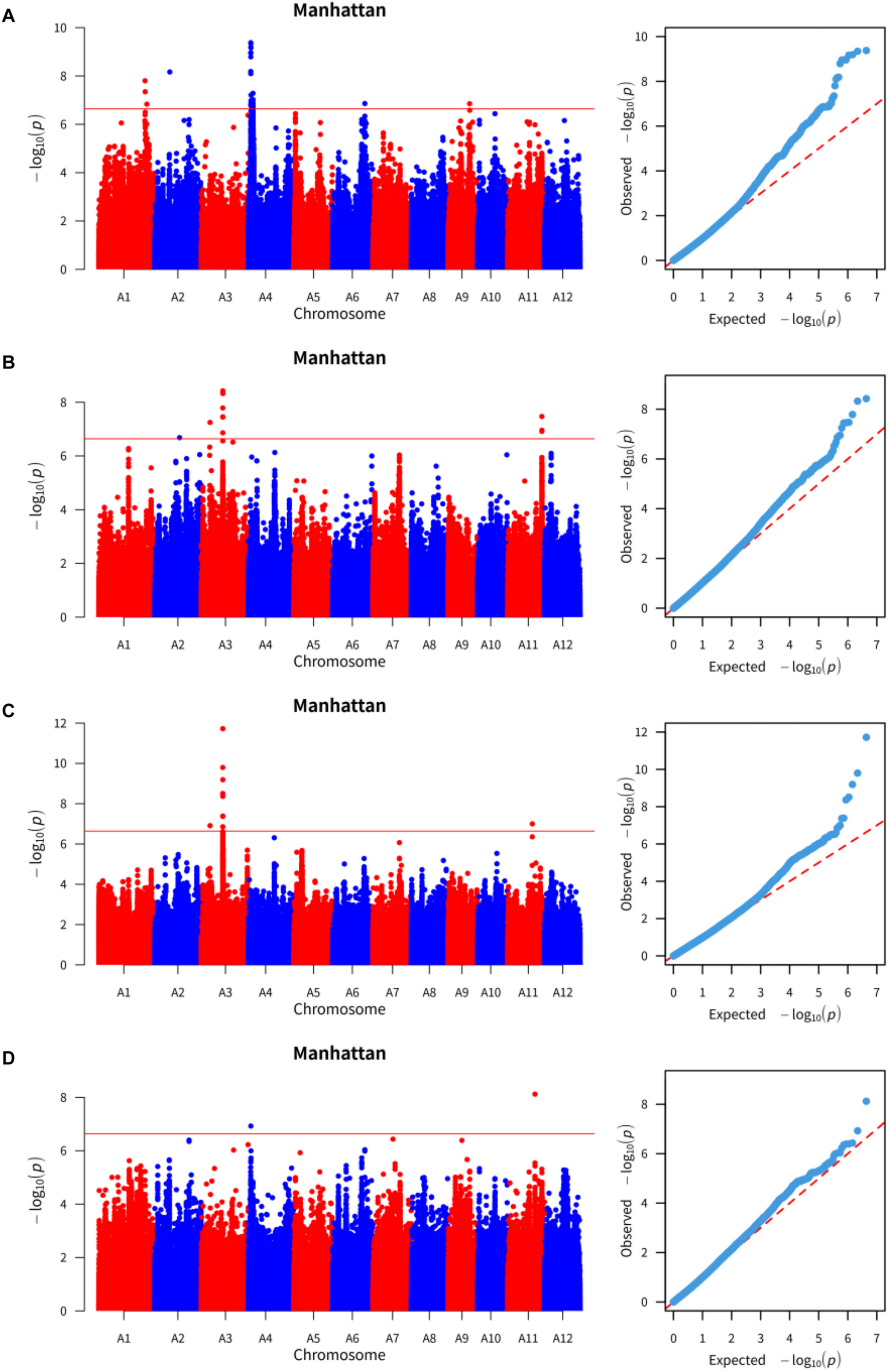
Manhattan plot (left) and quantile plot (right) of the GWAS results. (A) Grain length. (B) Panicle grain number. (C) Grain width. (D) Panicle length.

A GWAS was conducted using rice panicle traits as the phenotypic dataset. A total of 47 SNPs that were primarily located on chromosome 4 and were significantly associated with grain length were screened via the GWAS (Fig. 10).

The GWAS revealed 10 SNPs significantly associated with grain width, with the majority located on chromosome 3. In addition, 11 SNPs significantly associated with the number of grains per panicle were found, predominantly on chromosomes 3 and 11. For panicle length, 2 SNPs were identified, one each on chromosomes 4 and 11.

By examining regions 100 kb upstream and downstream of the significant SNPs using rice genome annotation files, we identified several genes previously reported to be significantly related to rice yield. Cytochrome P450 genes were mapped to the traits of grain width and grain number per panicle [25]. The ES2 gene, which is known to regulate rice leaf senescence and thus impact agronomic and yield traits [26], is linked to the panicle grain number. The panicle length and grain length traits were subsequently mapped to the CTB4a gene [27]. Observations and counts of the near-isogenic line NIL1913 containing CTB4a indicated its potential to enhance cold tolerance during the booting stage and improve pollen fertility under low-temperature conditions, thereby increasing the seed setting rate and yield. The grain length trait was mapped to the CTB2 gene, which confers strong cold tolerance and allows rice cultivation to expand from lower to higher altitudes and latitudes, notably to the colder regions of northeastern China, playing a significant role in the development of rice in cold areas [28]. In addition, we searched for MADS transcription factors, GS3, ATPase, translationally controlled tumor proteins and other genes that regulate rice growth and development and genetic variation.

## Discussion

### Reasons for grain width detection low accuracies

The extraction for grain width exhibited a relatively low *R*^2^ of 0.28, with an MAPE of 8.78% and an accuracy of 91.56%. Several factors may contribute to this observation. First, both the true and predicted grain width values are predominantly concentrated within the range from 2 to 3.3 mm. Although the differences between the predicted and true values are minor, resulting in minimal absolute error, the low variability within this narrow range may lead to a reduced *R*^2^, which indicates a weaker correlation. Second, the predicted grain width tends to be lower than the true value. This pattern suggests that the segmentation model used in this study, which relies on the location information from the grain counting model, may not consistently encapsulate the entire grain. The occurrence of incomplete or partially occluded grains can lead to an underestimation of the grain width. Despite efforts to adjust the IoU and Conf to ensure more complete grain images, the residual error from the grain counting model persists. This limitation indicates that the anchor frame might not always fully cover the grain, affecting the accuracy of the width measurement. Finally, the direct extraction of rice panicle traits without artificial shaping means that variations in the grain deflection angle can contribute to lower predicted values of grain width. A change in the angle at which a grain is imaged can alter its apparent width in the image, leading to inaccuracies.

### Analysis of reduced accuracies in field experiments

In our evaluation of EOPT, for rice varieties from various years, we observed decreases in the accuracy of the grain length and width measurements of 3.52% and 8.31%, respectively. When using images in the field for validation, the accuracies of the grain length and panicle length measurements were reduced by 4.27% and 7.34%, respectively, compared to the results obtained from the indoor validation. The observed decrease in measurement accuracy can be attributed to several factors, including varietal differences and the imaging environment. In this study, the threshold segmentation of rice grains was based on the red–green–blue (RGB) color range characteristics of the grains. Certain rice varieties exhibit distinct color ranges, leading to segmentation challenges. The reliance on a fixed RGB color range for segmentation does not account for the diversity across all rice varieties, which, in turn, affects the accuracy. The imaging environment and lighting conditions also play a critical role in threshold segmentation. In addition, the panicles are more dispersed when images are acquired indoors than when images are acquired outdoors. Less occlusion improves the accuracy of indoor shape extraction.

The reduction in panicle length measurement accuracy by 7.34% using EOPT is attributed to the method used to maintain consistency between the indoor and outdoor images. In the process of acquiring field images, manual occlusion was used to obscure a segment of the panicle, which was then excluded from the total measured panicle length. This practice resulted in nonstandardized starting points for the calculation of panicle lengths. The variability introduced by the manual exclusion of the obscured panicle segment, which could occur at any given position, may contribute to discrepancies between the intended cut position and the actual obscured region. Such inconsistencies are likely to compromise the accuracy of the panicle length measurements.

### The extended study of GWAS results

The EOPT was utilized to extract phenotypes from a cohort of 420 rice varieties. GWAS identified several genes implicated in the regulation of rice yields, such as *OsDWARF4*, *ES2*, and *CTB4a*. These genes have been reported in different studies over various years, reinforcing the reliability of our findings. The identification of functional variations in these genes contributes to our understanding of the genetic underpinnings responsible for the natural variability of rice yield traits.

By elucidating the genetic factors influencing yield traits, our findings may inform breeding programs and genetic engineering approaches. Furthermore, the methods and insights gained from this work have the potential to inform phenotype extractions in other crop species. The approach demonstrated by EOPT in rice could be adapted to facilitate similar studies in different agricultural contexts, thereby advancing the field of crop science.

### Scalability of EOPT

The processing time of EOPT was investigated by assessing a sample of 100 randomly selected panicle images. The results indicated that EOPT required 1.61 s to extract panicle lengths, 0.93 s for grain counting, and 1.15 s for grain length and width extractions per image. While this study primarily concentrated on specific traits, including the panicle grain number, panicle length, grain length and width, it is important to acknowledge the broader rice yield potential traits, such as the grain-setting rate and thousand-grain weight. These aspects represent expanded research avenues for EOPT.

The development of the grain counting model within this research utilized the PyTorch framework, providing a robust foundation for model training. Moreover, the deployment of the EOPT model spans various platforms, including cloud, computer, and mobile devices, indicating its versatility in supporting rice phenotyping tasks. Notably, there are scenarios where it is imperative to measure traits on intact rice panicles. For instance, circadian rhythms or growth and development patterns during different stages of rice cultivation have been studied. In this regard, EOPT demonstrates high potential due to its adaptability to diverse research needs and its ability to ensure accurate measurements without compromising the integrity of rice samples. In addition, the traits that EOPT is currently able to measure are not perfect. Accurate measurement of the seed setting rate and panicle structure in intact panicles is an important area of extension research for this study.

## Conclusion

This study introduced EOPT, an innovative method for the nondestructive extraction of intact rice panicle traits from images. By utilizing specialized software, EOPT simplifies the phenotyping process by eliminating the need for manual threshing and reshaping of rice panicles. We presented the PMI as a novel metric to quantify the degree of mutual occlusion between rice grains within a panicle, which significantly enhanced the panicle grain number determination accuracy. EOPT has demonstrated rapid and precise extraction capabilities for critical panicle traits, achieving measurement accuracies of 93.57% for grain number, 96.83% for grain length, 91.56% for grain width, and 97.13% for panicle length. Furthermore, its effectiveness has been validated across multiple cultivation years and environments, including for both indoor and field settings. The traits extracted from EOPT were used in a GWAS, leading to the identification of numerous previously reported genes. This finding not only underscores the reliability of EOPT but also highlights its broad applicability in genetic research. The approach advocated in this study not only facilitates the rapid assessment of rice panicle traits but also has potential for adaptation in the field. This offers a perspective and methodology for the investigation of crop yield, with implications extending beyond rice to other crops, paving the way for advancements in agricultural science and breeding strategies.

## Data Availability

The rice images and measured data in this study are available upon request by contacting the first of the corresponding authors. The link for software is https://pan.baidu.com/s/1BJ184slKAXJMwLmZznjRlA?pwd=6knk. The link for project is https://github.com/SUNJHZAU/EOPT.

## Supplementary Materials

Supplementary 1 Movie S1. Video of the Panicle Analyzer software.
